# CDC7 Expression in Selected Odontogenic Tumors

**DOI:** 10.1155/2022/6336003

**Published:** 2022-11-18

**Authors:** Zohreh Jaafari-Ashkavandi, Nahid Alizadeh

**Affiliations:** ^1^Department of Oral and Maxillofacial Pathology, School of Dentistry, Shiraz University of Medical Sciences, Shiraz, Iran; ^2^School of Dentistry, Shiraz University of Medical Sciences, Shiraz, Iran

## Abstract

**Objectives:**

CDC7 is a serine-threonine kinase that plays a key role in initiating DNA replication. It has been implicated in the growth and invasion of many pathologic lesions and suggested as a diagnostic marker. The aim of this study was to evaluate CDC7 in some odontogenic tumors.

**Materials and Methods:**

In this cross-sectional study, 45 cases, including 19 ameloblastomas, 15 dentigerous cysts, 7 ameloblastic fibromas, and 4 adenomatoid odontogenic tumors (AOT), were studied immunohistochemically. ANOVA and post hoc methods were used for statistical analysis.

**Results:**

CDC7 expression was observed in 93% of tumors and all dentigerous cysts. The expression rate was low. The results showed a higher expression rate of CDC7 in ameloblastoma and ameloblastic fibroma compared to AOT (*p*=0.009 and *p*=0.048, respectively). Ameloblastoma and ameloblastic fibroma were not significantly different in CDC7 expression (*p*=0.6).

**Conclusion:**

According to the results, the expression of the CDC7 protein in odontogenic tumors is low. The higher expression of CDC7 in ameloblastoma and ameloblastic fibroma in comparison with AOT confirms the hamartomatous growth of the latter, so it can be considered as a potential diagnostic marker. Future studies with a larger sample size are suggested to obtain a cut-off point for diagnostic purposes.

## 1. Introduction

Odontogenic tumors are rare neoplasms of the jaw that originate from the proliferation of odontogenic epithelium, ectomesenchyme, or both. Most of them are benign; however, they can be important due to invasive growth or recurrence [1]. Ameloblastoma is the most common odontogenic tumor with clinical significance. This tumor is benign, with slow-growing and locally invasive behavior and a high tendency to recur [2]. Solid and unicystic variants of ameloblastoma are the main types of this tumor with different clinical courses and management, so unicystic ameloblastoma is considered a less aggressive subtype with a low recurrence rate [3]. The luminal type of unicystic ameloblastoma presents and develops as a cyst and is considered in the differential diagnosis of the dentigerous cyst, and is managed similarly [4]. Adenomatoid odontogenic tumor (AOT) and ameloblastic fibroma are also rare and benign epithelial odontogenic tumors that are noninvasive, grow slowly, and usually have no recurrence [5]. These tumors have different biological behaviors and histopathologies. However, in some cases, they are considered in the differential diagnosis, and it is difficult to differentiate them even histopathologically. The mechanism of growth and invasion of these lesions is not exactly understood [2]. Today, immunohistochemistry (IHC) is used to understand the molecular basis and mechanism of progression and treatment of pathologic lesions [6]. This method is currently used for diagnostic and even therapeutic purposes, especially for lesions with similar or overlapping histopathological features as well as small or incisional biopsy specimens before making the final diagnosis. The assessment of the proliferation rate plays a critical role in diagnosing, managing, and even predicting the outcome of patients; hence, discovering new biomarkers and proliferation markers is an imperative need [7].

CDC7 is a serine-threonine kinase that is essential for the initiation of DNA replication. This protein is very rare or undetectable in normal tissues [8]. CDC7 is activated by Dbf-4 and Drf-1 proteins. These proteins bind together and activate CDC7, which causes phosphorylation at various sites on the MCM-2 protein. It is shown that phosphorylation of MCM 2 and other MCM 2–7 family proteins by CDC7-Dbf4 at the G1-to-S stage is critical for initiating DNA replication [9]. MCMs are the proteins involved in regulating DNA replication. These proteins ensure that DNA duplication occurs only once in each cell cycle [10]. They are standard markers of cell division and are commonly used to estimate the growth rate of cell populations in normal or neoplastic tissues. Because the increased expression of these proteins has been confirmed in various malignant tumors, they have diagnostic and prognostic values [11]. Martin et al. stated that CDC7 activity is required for the completion of DNA replication and that its inhibitor may be a potential therapeutic target for Ewing sarcoma [12]. Increased CDC7 expression has been observed in primary malignant tumors such as breast, colorectal, and also head and neck cancers, which has led to this protein being considered as a therapeutic target [13, 14]. CDC7 was a potential biomarker in the early diagnosis of cervical cancer, and inhibition of its expression could prevent the proliferation and migration of cervical cancer cells and promote apoptosis [14]. It has been found that increased CDC7-dependent replication is a hallmark of the P53 mutation, and the knockdown of Cdc7 expression induces p53-independent apoptosis, suggesting that Cdc7 is a target for cancer treatment [15, 16]. Moreover, in a previous study, we showed an increased CDC7 expression rate in odontogenic keratocyst (OKC), which are locally aggressive odontogenic cysts, in comparison with dentigerous cysts [17]. The aim of this study was to evaluate the expression rate and usefulness of CDC7 in the differential diagnosis of some odontogenic tumors. So far, there has been no available data about the expression of this marker in odontogenic tumors.

## 2. Materials and Methods

This research was approved by the Medical Ethics Committee of Shiraz University of Medical Sciences (IR.SUMS.REC. 1395.S1015). In this retrospective and cross-sectional study, 19 cases of ameloblastoma (12 solid and 7 unicystic), 4 cases of AOT, 7 cases of ameloblastic fibroma, and 15 cases of a dentigerous cyst (just for comparison with luminal ameloblastoma) were evaluated immunohistochemically. The cases were collected from the archive of the oral and maxillofacial pathology department at Shiraz University of Medical Sciences. All cases with a definitive diagnosis and a sufficient epithelial component were enrolled. Clinical data, including the patients' age and gender, as well as the location of tumors, were noted according to the patients' medical files. The study was conducted according to the Declaration of Helsinki, and all patients signed informed consent forms.

IHC staining was performed using the Envision Labeled Peroxidase System (DAKO, Carpentaria, CA, USA). 4 *μ* sections were prepared from formalin-fixed and paraffin-embedded specimens. After deparaffinization in xylene, the sections were washed in dehydrated alcohol and distilled water. Antigen retrieval was performed by DAKO Cytomation solution with pH = 9 for 20 minutes. Endogenous peroxidase activity was inhibited by a 3% H_2_O_2_ solution. The sections were incubated with primary CDC7 polyclonal antibody (1 : 50 dilution, Gentex, USA) for 60 min. The envision system was applied as the secondary antibody, and the sections were washed in phosphate-buffered saline (PBS). Finally, the sections were stained with 3,3′-diaminobenzidine tetrahydrochloride as a chromogen and counterstained with Mayer's hematoxylin.

Basal cells of the oral mucosa were used as the positive control, and the primary antibody was replaced by PBS as the negative control. Cells with brown nuclei were considered to have positive CDC7 staining. At least 300 tumoral cells were counted in each specimen, and the percentage was noted. In the luminal ameloblastomas and dentigerous cysts, the basal and parabasal cells were evaluated. Samples with a staining percentage of <5% were considered to have low CDC7 expression, those between 5–10% as moderate expression, and those >10% as high CDC7 expression. Data were analyzed using SPSS software version 11. ANOVA and post hoc methods were used for statistical analysis. *p*-values less than 0.05 were considered statistically significant.

## 3. Results

45 specimens were studied. The patients were 10–63 years old with a mean age of 27.4 ± 13.5. Baseline data regarding patients' age and gender, as well as the location of tumors, are shown in Table 1.

**Figure 1:**
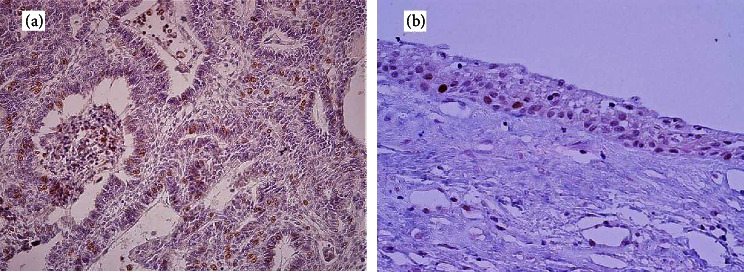
(a) Nuclear CDC7 expression in ameloblastoma (×200); (b) nuclear CDC7 expression in the basal cell layer of a dentigerous cyst (×400).

Nuclear CDC7 expression was observed in 80% of tumors. CDC7 was expressed in 79% of the ameloblastomas. Marker expression was observed in the epithelial cells, especially in the peripheral ameloblastic cells, but not in the tumor stroma (Figure 1(a)). In the limited cases of unicystic ameloblastomas, the luminal types showed a minimum level of CDC7 expression (about 1%), and in mural ameloblastoma, CDC7 was found in the epithelial cells of the invaded ameloblastic nests, similar to the solid ameloblastomas. In dentigerous cysts and luminal ameloblastomas, the expression was low and limited to the basal cell layer (Figure 1(b)).

**Figure 2:**
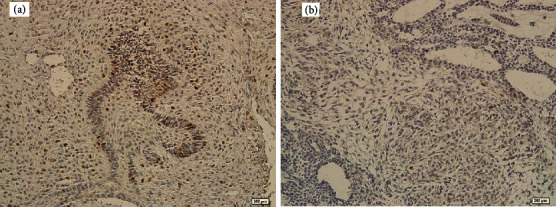
(a) Nuclear CDC7 expression in ameloblastic nests and stroma of ameloblastic fibroma (×200); (b) low and weak nuclear CDC7 expression in AOT (×200).

In 86% of ameloblastic fibromas, CDC7 has been expressed in the ameloblastic nests as well as the stromal mesenchymal cells (Figure 2(a)). In 75% of AOTs, tumoral epithelial cells showed CDC7 expression with very low intensity (Figure 2(b)). The mean of CDC7 expression and its classification are illustrated in Tables 2 and 3. According to the tables, the expression of the CDC7 protein was low in a large percentage of the studied tumors. The result of the ANOVA test showed a significant difference in the mean of CDC7 expression among the groups (*p*=0.03). According to the post hoc (Tukey) test, there was no significant difference in CDC7 staining between ameloblastoma and ameloblastic fibroma (*p*=0.36); however, this test showed that CDC7 expression in AOT was significantly lower than that of ameloblastoma (*p*=0.009) and ameloblastic fibroma (*p*=0.04). Due to the limited number of ameloblastoma subgroups, no statistical analysis was performed.

The stromal mesenchymal cells in ameloblastic fibroma revealed nuclear CDC7 expression; however, in the other lesions, the expression was limited to some inflammatory or a few endothelial cells (Figures 1 and 2).

## 4. Discussion

Since CDC7 is considered a therapeutic target in some tumors, and so far, no research has been done on the significance of the expression of this marker in odontogenic tumors, in the present study, the expression of CDC7 has been investigated in a group of more prevalent and challenging odontogenic tumors.

In this study, 80% of tumors, including ameloblastoma, ameloblastic fibroma, and AOTs, showed very low CDC7 expression, with a mean of 7%. Several studies have evaluated the expression of CDC7 in benign tumors. Huggett et al. showed that CDC7 expression in benign pancreatic tumors was low, with a mean of 1.3% [18]. Clarke et al. found that CDC7 expression in benign cutaneous melanocytic lesions was less than 1% [19]. In a previous study, we detected CDC7 in 3.5%–20% of the basal cells in some odontogenic cysts. The higher rate was related to odontogenic keratocysts with more aggressive behavior [17].

In the present study, despite the low protein expression, ameloblastoma is an invasive benign tumor, and ameloblastic fibroma showed higher CDC7 expression than AOT, which develops hamartomatous. These findings are in line with those of Razavi et al. in comparing the expression of Bcl2 and Ki67 markers between solid ameloblastoma and AOT. They showed that although the expression level of Ki67 in solid ameloblastoma was low, it was higher than that in AOT. This finding confirms the aggressive behavior of solid ameloblastoma as well as the hamartomatous nature of AOT [20]. Also, one study revealed a significantly higher Bcl-X expression in ameloblastoma in comparison with AOT, which could be suggestive of a difference in the growth profile and aggressiveness of these odontogenic tumors [21]. Lee and Kim also reported higher expression of cell proliferation markers in ameloblastoma than AOT [22]. CDC7 plays a key role in initiating DNA replication and the S/G1 phase (cell cycle checkpoint). CDC7 phosphorylates the MCM2-7 complex. Activation of the internal pathway activates DNA-helicase activity and begins DNA transcription, thus increasing cell proliferation [23]. In normal cells, a P53 gene-dependent pathway actively inhibits the progression of the S phase in the absence of sufficient CDC7 kinase and arrests the cell cycle in the G1 phase [13]. Datta et al. [15] demonstrated that an increase in the initiation of CDC7‐dependent replication was a hallmark of p53 gain‐of‐function mutations. In previous studies, AOT showed a weaker expression of p53 in comparison with ameloblastoma [24]. Also, Anjum et al. in 2016 stated that increased MCM2 expression in ameloblastoma might cause local invasion and recurrence of this tumor [25]. It is suggested that lower expression of Ki67 in ameloblastic fibroma might be a factor in the less aggressive behavior and slower growth of this tumor in comparison with ameloblastoma [26].

In this study, CDC7 expression was not observed in the stroma of ameloblastomas, but in ameloblastic fibroma, the stromal cells also showed the expression. This indicates the activity of the stromal cells in ameloblastic fibroma and can be further investigated as an aid in differentiating this tumor from other odontogenic tumors. There have been few IHC investigations on ameloblastic fibroma. Some researchers evaluated highly aggressive ameloblastic fibromas to rule out malignant transformation and reported the Ki67 marker in the epithelial and mesenchymal components of tumors, as well as P53 in the epithelial component [27]. Moreover, postnatal mesenchymal stem cells have been found in the dental pulp and some odontogenic tumors such as ameloblastic fibroma, with the ability to proliferate and differentiate, and they are currently considered for disease management and regenerative therapy [28–30]. The presence of these cells may be related to the proliferation and invasion of tumors.

Unicystic ameloblastoma may be a challenging diagnosis in oral pathology, which can be considered in the differential diagnosis of dentigerous cysts, especially in small biopsy specimens. The present findings did not show any significant difference between a few cases of luminal unicystic ameloblastoma and a dentigerous cyst. A previous study also reported a similar expression of Ki67 and MCM3 proliferation markers in a limited number of unicystic ameloblastomas in comparison with dentigerous cysts [31]. Both of the lesions have slow growth and no tendency toward recurrence [4]. However, further examination is required to investigate the difference between unicystic ameloblastoma and dentigerous cysts. Data about most of the rare odontogenic tumors are limited in the literature, and this study was an attempt to investigate the usefulness of novel proliferation markers in these tumors. However, there were some limitations. The low number and small sample size of specimens that are usually submitted to oral pathology departments make it difficult to carry out more comprehensive research on these tumors. However, this study could provide information as a preliminary study for subsequent researches.

## 5. Conclusion

According to the results of this preliminary study, it can be concluded that the expression of the CDC7 marker in benign odontogenic tumors is low. The higher expression of CDC7 in ameloblastoma and ameloblastic fibroma in comparison with AOT might confirm the hamartomatous growth of the latter and can be considered as a potential diagnostic marker. Future studies with a larger sample size are suggested to obtain a cut-off point for diagnostic purposes and evaluate the exact use of this marker in the differential diagnosis of similar lesions.

## Figures and Tables

**Table 1:** Baseline data of all groups.

Groups (N)	Male: Female	Age mean ± SD	Maxilla: Mandible
Dentigerous cyst (15)	13 : 2	29.76 ± 10	6 : 9
Ameloblastoma (19)	8 : 11	30.3 ± 14 .9	1 : 18
Ameloblastic F (7)	3 : 4	21 .7 ± 8.1	2 : 5
AOT (4)	0 : 4	24.2 ± 12.7	1 : 3
Total (45)	24 : 21	28.5 ± 13.5	4 : 26

F, Fibroma; AOT, Adenomatoid odontogenic tumor.

**Table 2:** CDC7 expression rate in all groups.

Diagnosis	Mean of CDC7 (%)	Min-max (%)	*p*-value
Ameloblastoma	8.27 ± 7	1–30	0.009 vs. AOT
			0.6 vs. ameloblastic F.
Solid ameloblastoma	8.7 ± 4.8	1–30	
Unicystic ab (mural)	6.46 ± 4.69	1–20.3	
Unicystic ab (luminal)	1.2 ± 1	1–3	
Ameloblastic F	3.71 ± 3.25	1–10	0.6 vs. AB
			0.04 vs. AOT
AOT	0.76 ± 0.48	0–1	0.009 vs. AB
			0.04 vs. ameloblastic F.
Dentigerous cyst	3.5 ± 1.4	1–6.1	
Total	7.08 ± 5.04	0–30	

ab, Ameloblastoma; *F*, Fibroma; AOT, Adenomatoid odontogenic tumor.

**Table 3:** The status of CDC7 expression in all groups.

Group	Expression status		
	Low (%)	Moderate (%)	High (%)
Ameloblastoma	10 (52.6)	4 (21.1)	5 (26.3)
Ameloblastic fibroma	4 (57.1)	2 (28.6)	1 (14.3)
AOT	4 (100)	0	0
Dentigerous cyst	13 (86.6)	2 (13.3)	0
Total	31 (68.9)	8 (17.8)	6 (13.3)

AOT: Adenomatoid odontogenic tumor.

## Data Availability

The data are available from the corresponding author upon request.
